# Novel Algorithm for Radon Real-Time Measurements with a Pixelated Detector

**DOI:** 10.3390/s22020516

**Published:** 2022-01-10

**Authors:** Alessandro Rizzo, Francesco Cardellini, Claudio Poggi, Enrico Borra, Luca Ciciani, Livio Narici, Luciano Sperandio, Ignazio Vilardi

**Affiliations:** 1 Radiation Protection Institute (IRP)—Italian National Agency for New Technologies, Energy and Sustainable Economic Development (ENEA), Via Anguillarese 301, 00123 Rome, Italy; enrico.borra@enea.it (E.B.); luca.ciciani@enea.it (L.C.); luciano.sperandio@enea.it (L.S.); Ignazio.vilardi@enea.it (I.V.); 2 National Institute of Ionizing Radiation Metrology (INMRI)—Italian National Agency for New Technologies, Energy and Sustainable Economic Development (ENEA), Via Anguillarese 301, 00123 Rome, Italy; francesco.cardellini@enea.it; 3 Radiation Protection Institute (IRP)—Italian National Agency for New Technologies, Energy and Sustainable Economic Development (ENEA), Via Enrico Fermi 45, 00044 Rome, Italy; claudio.poggi@enea.it; 4 Physics Department, University of Rome “Tor Vergata”, Via Della Ricerca Scientifica 1, 00133 Rome, Italy; narici@roma2.infn.it

**Keywords:** radon, real-time measurements, TimePix, MediPix, pattern recognition algorithm

## Abstract

Nowadays, radon gas exposure is considered one of the main health concerns for the population because, by carrying about half the total dose due to environmental radioactivity, it is the second cause of lung cancer after smoking. Due to a relatively long half-life of 3.82 days, the chemical inertia and since its parent Ra-226 is largely diffuse on the earth’s crust and especially in the building materials, radon can diffuse and potentially saturate human habitats, with a concentration that can suddenly change during the 24 h day depending on temperature, pressure, and relative humidity. For such reasons, ‘real-time’ measurements performed by an active detector, possibly of small dimensions and a handy configuration, can play an important role in evaluating the risk and taking the appropriate countermeasures to mitigate it. In this work, a novel algorithm for pattern recognition was developed to exploit the potentialities of silicon active detectors with a pixel matrix structure to measure radon through the *α* emission, in a simple measurement configuration, where the device is placed directly in air with no holder, no collection filter or electrostatic field to drift the radon progenies towards the detector active area. This particular measurement configuration (dubbed as *bare*) requires an *α*/*β*-discrimination method that is not based on spectroscopic analysis: as the gas surrounds the detector the *α* particles are emitted at different distances from it, so they lose variable energy amount in air depending on the traveled path-length which implies a variable deposited energy in the active area. The pixels matrix structure allows overcoming this issue because the interaction of *α*, *β* and *γ* particles generate in the active area of the detector *clusters* (group of pixels where a signal is read) of different shape and energy dispersion. The novel algorithm that exploits such a phenomenon was developed using a pixelated silicon detector of the TimePix family with a compact design. An *α* (Am-241) and a *β* (Sr-90) source were used to calibrate the algorithm and to evaluate its performances in terms of *β* rejection capability and *α* recognition efficiency. Successively, the detector was exposed to different radon concentrations at the ENEA-INMRI radon facility in ‘bare’ configuration, in order to check the linearity of the device response over a radon concentration range. The results for this technique are presented and discussed, highlighting the potential applications especially the possibility to exploit small and handy detectors to perform radon active measurements in the simplest configuration.

## 1. Introduction

Radon (Rn-222) is a naturally occurring radioactive gas produced by the decay of the Ra-226 radionuclide which is largely diffused on the earth’s crust as well as in building materials, as it belongs to the natural U-238 chain. The large occurrence of the parent radionuclide Ra-226 in indoor and outdoor environments, combined with the radon half-life of 3.82 days and its chemical inertia, allows this noble gas to travel for long distances from the emanation point and potentially saturate human habitats as houses and workplaces exposing the population to potentially high-risk.

The inhalation of radon, and in particular of its *α* emitters daughters like Po-218 and Po-214, is one of the main causes of lung cancer due to the high ionizing power of the emitted *α* particles which can damage cells and DNA inside the nucleus [1,2,3,4]. For these reasons, the World Health Organization (WHO) considers radon gas as one of the main health concerns among those related to environmental radioactivity exposure [5,6], carrying almost half the total dose due to environmental radiation. Therefore, radon activity concentration measurements play a fundamental role to evaluate the exposure risk.

The main techniques used to monitor radon activity concentration in air are two: (i) integration techniques, generally used by passive detectors like dosimeters or electrets [7,8,9,10], which return the integral value of radon concentration measured over the time period of the detector exposure and (ii) real-time techniques, performed by *active* detectors, that perform radon concentration measurements on small pre-set time intervals, that can be repeated over the total time of exposure. The large use of passive detectors, due to their low cost and simple usage, affected until now both the risk evaluation and the radiological protection limits (both referred to mean concentration values on long periods, generally one year), but according to the present knowledge of the indoor radon diffusion [11,12,13,14], the gas concentration shows a large variability between day/night time with evident seasonal variations. Hence, real-time measurements of radon concentration in air assume a fundamental importance to monitor the exposure variation over the proper timescale, allowing also to promptly undertake the appropriate countermeasures to mitigate the risk.

Among the existing *active* detectors (for example, Lucas cells [15,16], Ionization chambers [17,18] with free or forced air circulation), it is important to identify new detectors that can profit from small dimensions and simple usage. The small dimensions, in particular, combined with the intrinsic capability of the active measurements to overcome the air turbulences problem, which affects the use of integration techniques [19], allow a measurement of the ‘local’ value of radon concentration in human habitats and the radon indoor distribution to be reconstructed. In this work, a Timepix detector, originally designed to monitor the ionic radiation environment inside human space habitats, was used to perform radon real time-measurements. Devices of this family have been already used for this kind of measurement, as presented in the literature for the RADONLITE and RADONPIX detectors [20] and in the setup reported in [21], which employ *α* spectroscopy techniques to detect and identify Po-218 and Po-214 radon daughters. In the first approach, the positive ions of Po-218 and Po-214 were concentrated onto the TimePix active area using an electrostatic field generated inside a custom-made detector holder. The *γ* and *β* background was rejected since the *α* particles deposited larger energy in silicon. In the second work, the TimePix is coupled to a filter where the air and so radon and its progenies are flushed and *α* particles discrimination is based on spectroscopic methods.

In the work presented here, a new approach is proposed to use the TimePix detector for radon concentration measurements in the simplest configuration (hereafter referred to as ‘bare’ configuration), where the detector is placed directly in the air with no holder, no filter, and no external drift field to catch radon progenies. In this configuration, the challenge in *α* discrimination is the variability of the deposited energy onto the active area of the detector, a consequence of the different energy lost by ionization in air due to the different distance that can occur between the emitting atom of the gas and the detector. For this reason, the *α* discrimination technique can not rely upon spectrometric methods, but a different approach is necessary. Since *α*, *β*, and *γ* particles produce different signatures in semiconductor detectors, these are seen in a silicon pixels matrix structure as clusters (group of pixels with a signal due to the incident particle interaction) with different shape end energy dispersion around the seed (pixel with the maximum deposited energy), independently from the deposited energy. A specific pattern recognition algorithm was developed to recognize *α* particle tracks, by defining two specific variables: the *RoundnessR*, related to the cluster geometry and the *Core energy* *C*, related to the deposited energy spread around the seed. The number of recognized *α* particles is then used, in radon exposures, to evaluate the *α* counts per minute (cpm) and correlate it with the radon activity concentration.

## 2. Materials and Methods

### 2.1. The TimePix Detector

The Timepix detector used in this work is a silicon pixel sensor developed by the Medipix2 collaboration at CERN [22,23] and specifically designed, in a small and handy configuration, for space-applications by a collaboration between NASA and the University of Houston. The Silicon chip and associated electronics (read-out and power-supply) are housed in a USB-stick [24], with a plug-and-play configuration, that can easily be connected with a computer for acquiring data. In such configuration, this detector is largely used to monitor the radiation environment inside the International Space Station (ISS) [25] and its usage is also planned for the future Orion Exploration Flight Test-1 (EFT-1) in lunar orbit [26].

**Figure 1:**
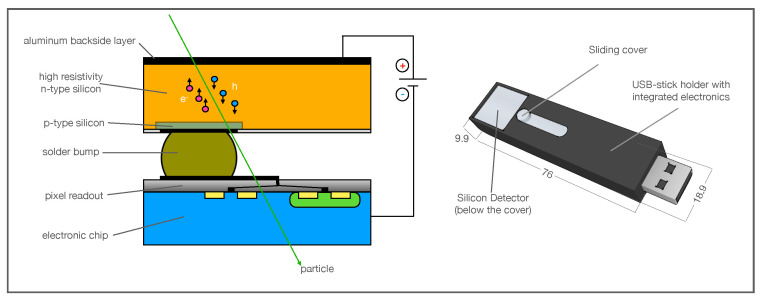
Left Canvas: the sketch shows the vertical cross-section of the Timepix detector. The p-type superficial implantation on the n-type semiconductor creates a local depletion zone corresponding to the single pixel. The pixel is then connected to the ASIC read-out chip through a solder bump. Right Canvas: Sketch of the TimePix detector. The dimensions are in mm.

The detector consists of a semiconductor sensor (size 14 × 14 mm^2^, 300 μm thick) bump-bonded to an ASIC readout chip of 256 × 256 pixels with a pitch size of 55 μm [27]. The matrix structure is obtained in the silicon sensor, which is an n-type semiconductor wafer, with insertion of superficial p-type contacts in a matrix configuration that locally generates a depletion region (V_bias_ ∼ 100 V) corresponding to the active area of a single pixel (Figure 1, left canvas).

The holes generated by the ionization process are collected towards the p-type insertion read by the ASIC chip through the bump-bonded contact [28]. The signal induced by the charge collection can be processed by the read-out electronics in *counting mode*, in *energy mode* or in *time mode* [21]. In this work, we operated the detector in *energy mode*, a configuration based on the Time-over-Threshold (ToT) acquisition, where for each pixel the ADC counter is incremented continuously as long as the signal induced by the particle is above a certain threshold. As the ToT is proportional to the charge collected by the preamplifier, the ADC value is linearly dependent on the energy deposited into the pixel by the detected particle. Each pixel of the matrix is read simultaneously in a pre-set integration time which starts from 10 μs and the data are acquired by the Pixet software [24] installed on the computer that the detector is connected to.

### 2.2. The ENEA-INMRI Radon Facility

The radon data were acquired by the Timepix detector in ‘bare’ configuration exposing it in the ENEA-INMRI radon facility, where the gas activity concentration in air can be set and controlled (for a detailed description see [29]). The exposure chamber of the facility has a total volume of 150 m^3^ and is located on the ground floor of the laboratory in order to exploit the natural U-238 and Th-232 richness of the building basement materials. The radon formed in the basement is carried in the facility room through six vertical pipes drilled in the chamber floor. The gas can diffuse naturally, reaching low activity concentration values, or can be diffused at high activity concentration by lowering the pressure inside the building through a forced air-circulation system. The radon concentration values can span in this way the range from 0.1 to 18 kBq/m^3^ with good spatial uniformity throughout the chamber. On the other hand, the thoron (Rn-220) concentration is much less uniform as its short half-life of less than a minute prevents it from completely diffusing through the chamber volume. Two calibrated AlphaGUARD PQ 2000 PRO Professional Radon Monitors (Genitron, Germany) [30] are used to measure and control the radon concentration, temperature, pressure, and relative humidity in the chamber. The Airborne Radon Decay Products (RDPs) concentrations are monitored by a BWLM PLUS 2 S Radon Daughter Monitor (Tracerlab, Germany), which allows to measure the total airborne RDP (attached and unattached) and also only the attached fraction. As a consequence of the radon radioactive decay, the RDPs lose the initial chemical inertia of the noble gas tending to complete their external electron octet by attaching them to other atoms/molecules that can be found in the dust/particulate eventually present in air or on the walls/floor of the room. For this reason, the RDPs attached fraction concentration, which is the largest part, can be increased in a higher particulate concentration in air. A stearic candle burning is a technique generally used in the facility to raise the particulate concentration in air up to 10^5^ cm^−3^.

### 2.3. Algorithm for Discrimination of *α* and *β* Particles

**Figure 2:**
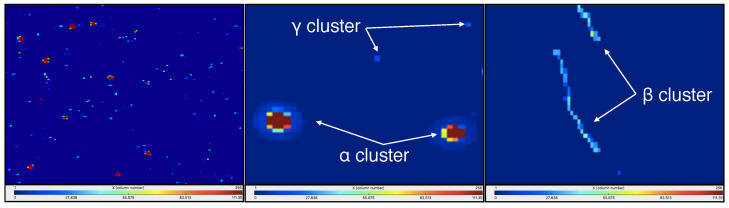
Left canvas: typical TimePix frame acquired for radon measurement in the ‘bare’ configuration (no holder). Other two canvas: clusters shape for α, β and γ particles interacting with Timepix detector. The clusters appear circular for α particles, dots for photons, and long tracks for electrons. The deposited energy is represented by the colored scale below the images.

The detector matrix structure allows to discriminate *α*, *β*, and *γ* particles interacting in the active area, given the different shapes of clusters generated by the different number and distribution of the electron-hole pairs created along the particle path. As shown in Figure 2, each type of particle can be associated with a different shape of cluster, in particular *α* clusters are almost circular, *β* clusters are linear or curly (*δ* rays) and clusters for *γ* particles are formed by one or two pixels.

Let’s consider for the following that the particles enter in the detector active area from the aluminum backside layer shown in the left canvas of Figure 1. An *α* particle track develops almost orthogonally to the detector plane, generating the highest number of electron-hole pairs along and symmetrically around the particle track in the detector, affecting several pixels where a signal is generated. Peripheral pixels in the cluster can be interested by image-charge induced signals due to the high ionization charge density collected in the neighboring pixels. Consequently, *α* cluster shows an almost circular shape with the largest part of deposited energy in the center of the cluster (that matches the seed) and in its first neighboring pixels. Differently, *β* particles are characterized by larger scattering angles in interaction with the medium, so, after the first scattering in the detector active area, the electron tracks can develop almost longitudinally with respect to the detector plane. The electrons tracks are characterized by multiple-scattering process with the electrons of the medium and low ionization power, resulting in the typical line or stripe shape (or curly ones if they are high-energy electrons) for *β* clusters, with an energy deposition that is almost homogeneous among the cluster pixels. Photons (*X* or *γ*) generate clusters composed by one or two pixels as the small detector thickness does not generally allow to contain the photo-extracted electrons [27] which deposit energy in only one or two adjacent pixels.

In order to exploit the capability of a pixelated detector, as the TimePix, to discriminate *α* particles to measure the radon, a specifically C++ designed algorithm based on ROOT libraries was developed to perform pattern recognition and discriminate the *α* particles for radon measurements. The algorithm was calibrated by exposing the detector to two certified planar sources of Am-241 (for *α* and *γ* particles) and Sr-90 (for *β* particles), with an activity of 1.04 (±6%) kBq and 0.84 (±6%) kBq respectively. The sources were placed in air, 5 mm far from the detector area, the acquisition rate was set to 0.33 Hz and a single-pixel energy threshold is fixed to 1 keV to reject electronic noise and simultaneously to accurately reconstruct the cluster. The chosen configuration allows a 256 × 256 matrix of values to be acquired avoiding frame overpopulation, which is unfit to tune the algorithm, keeping under control and substantially avoiding the cluster superimposition occurrence.

**Figure 3:**
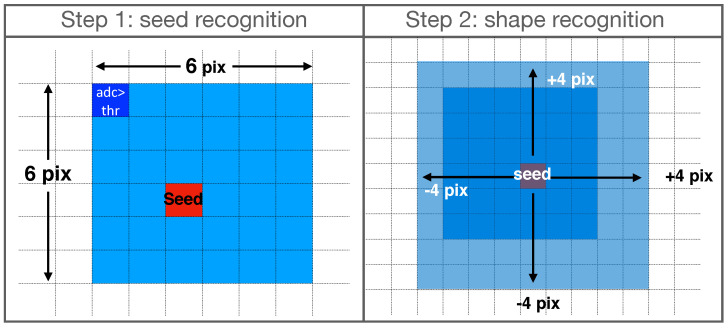
Pattern recognition algorithm sketch. The seed recognition part is shown in the left canvas. Once the deposited energy in a pixel is found to be above a certain threshold, a maximum search routine is implemented in a 6×6 area, in order to find the cluster seed. The shape recognition part, shown in the right canvas, starts from the recognized seed for a larger area, equal to 9×9 pixels. The deposited energy of each pixel in this area is summed up to calculate the cluster energy.

The pattern reconstruction consists of two steps (see Figure 3): the first one is the *seed* recognition, and the second is the shape recognition, which takes place around the identified seed. The silicon matrix is read from the first line (above) to the last line (below), from left to right. When the algorithm finds a pixel with a deposited energy value greater than a fixed threshold (generally set equal to 1 keV), a search area of 6 ×6 pixels is set, and a simple maximum search of deposited energy is performed in it. The 6 × 6 pixels square area was chosen because this value maximizes the algorithm *α* recognition efficiency (see Section 3.2).

Considering the *α* clusters central symmetry, a second step is performed in cluster reconstruction, considering a 9 × 9 pixel square, centered on the seed. This value, slighlty larger than a typical *α* cluster extension, permits to avoid the problem of the signal cut off when the cluster is reconstructed or when two clusters are close to each other. Each pixel with deposited energy greater than the threshold value inside this area is summed up to reconstruct the total energy deposited by the *α* particle in the cluster. For each cluster, the total number of pixels and the total energy are saved, flagging the read pixels to avoid double reading in the case of adjacent clusters. The 9 × 9 pixels square area was chosen as this value maximizes the *β* rejection capability of the algorithm (see Section 3.1).

**Figure 4:**
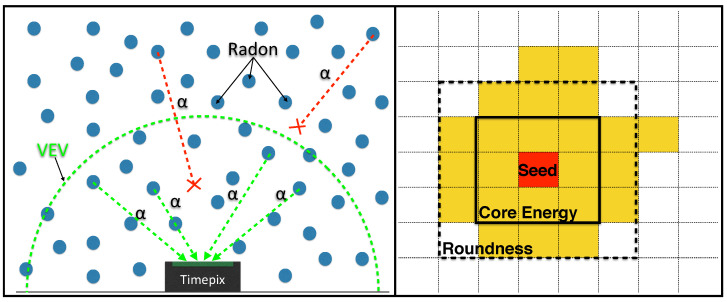
Timepix ‘bare’ configuration sketch for radon measurements (left canvas). The Virtual Enclosure Volume (VEV) surrounds the TimePix and includes all the atoms that can emit α particles capable to deposit some energy in the detector active area. For a given α particle energy, the maximum path-length traveled by the particle releasing an infinitesimal energy ϵ>0 in the silicon active area provides the volume dimension. In the right canvas a sketch of an α cluster, with the seed in red (color on-line) and the squares used to define variable C (3×3) and variable R (5×5).

As stated in the introduction, the information about the released energy by the *α* particles hitting the detector is not helpful alone to detect particles of interest when the measurement is performed directly in air in ‘bare’ configuration. As shown in the left canvas of Figure 4, the detector in ‘bare’ configuration is completely surrounded by radon gas and its progeny atoms, whose position with respect to the detector is fully random with a variable distance from the active area. Consequently, the path-lengths traveled by the *α* particles span from zero to a maximum value (as a function of the particle energy) implying different energy amounts lost in air ionization and consequently different amounts of deposited energy in the detector. In particular, considering that a 5 MeV *α* particle loses almost all its kinetic energy in a few centimeters of air in STP conditions [31], the variability of the *α* path length modifies drastically the expected energy spectrum, spreading the events forming expected *α* peaks all over the full accessible energy range. In order to consider this phenomenon, peculiar of the ‘bare’ configuration, it is useful to define a Virtual Enclosure Volume (VEV). This is the volume surrounding the detector for which the *α* particles emitted by atoms within it can reach the detector and release some energy in its active area. The dimension of such a volume depends on the maximum distance that an *α* particle can travel without losing all its energy by ionizing the crossed medium. As detailed in Section 3.4, such a distance was estimated by placing the Am-241 source at increasing distances and measuring the energy deposited in the detector, extrapolating the path-length value that corresponds to an infinitesimal deposited energy ϵ>0 in the detector.

The clusters discrimination step, that follow the cluster reconstruction, is based in this work on geometric topology, where two specifically defined control variables are used to disentangle *α* signatures: the *roundness* (*R*) and the *Core energy* (*C*) of the cluster on silicon active area. The *Roundness* variable, related to the cluster geometrical shape, is defined as:

(1) $$R=\sum_{□}N_{pix}$$

where the sum is over the pixels with a signal $N_{pix}$ enclosed in the black-dotted line square (□) represented in the right canvas of Figure 4. The square area, defined for this variable equal to 5×5 pixels, was chosen as the value that maximizes the beta rejection capability of the algorithm (see Section 3.1).

The *Core energy* variable for a given cluster, is related to the deposited energy dispersion around the seed and defined as:

(2) $$C=\frac{\sum_{▪}E_{dep}}{E_{cluster}}$$

where the ratio is between the sum over the deposited energy of the pixels enclosed in the black-continuous line square (▪ 3×3 pixels), represented in the right canvas of Figure 4, and the total deposited energy in the cluster evaluated in the 9 × 9 pixels area. A linear transformation (rotation) is applied to the (*R*,*C*) variables in order to obtain a single control variable useful to discriminate *α* clusters according with the equations:

(3) $$\left\{\begin{array}{c} R^{\prime }=R\cos\left(\theta \right)-C\sin\left(\theta \right)\\ C^{\prime }=R\sin\left(\theta \right)+C\cos\left(\theta \right) \end{array}\right.$$

The θ value is chosen as the one which maximizes the correlation between *R*’ and *C*’, allowing to use only the new variable *R*’ for *α* particle discrimination.

## 3. Results

### 3.1. Algorithm Optimization

In order to study the detector response and optimize the cluster discrimination algorithm, the TimePix was exposed to Am-241 and Sr-90 sources separately. For each source, 200 frames were acquired with an integration time of 3 s with a pixels threshold of 1 keV for rejecting possible electronic noise.

The linear correlation coefficient for the control variables R and C was evaluated considering separately data from Am-241 and Sr-90 sources, resulting in a slight correlation for *α* particle ($\rho_{\left(R,C\right)}=0.30$) and an almost null correlation for β particles ($\rho_{\left(R,C\right)}=-0.04$).

**Figure 5:**
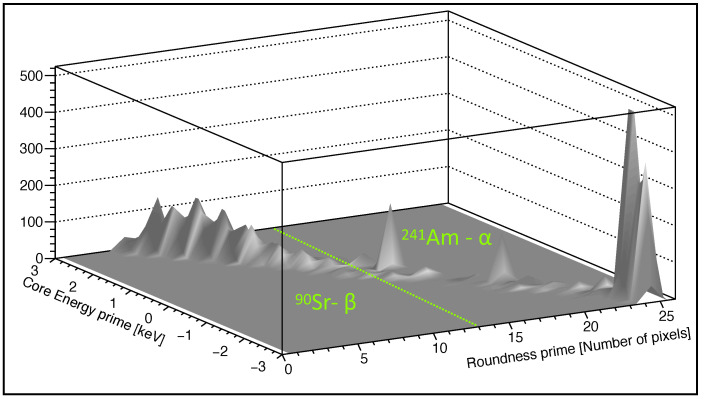
The three-dimensional plot (*R*’,*C*’) obtained for Am-241 and Sr-90 sources. α particles can be discriminated from β particles using a cut on *R*’ ≥13 as shown by the dotted line in figure.

Producing the three-dimensional plot (*R*’,*C*’) according to Equation (3) with $\theta =344^{\circ }$, separately for Am-241 and Sr-90 data, the α clusters are identified for a value of *R*’ ≥13, as shown in Figure 5. As previously stated, γ signatures are characterized by a single- or double-pixel clusters, so photons emitted by Am-241 (59.5keV) should fall in a region with a relatively small value of the *R*’ parameter, and are rejected automatically by the algorithm, requiring clusters with more than 3 pixels in total.

**Figure 6:**
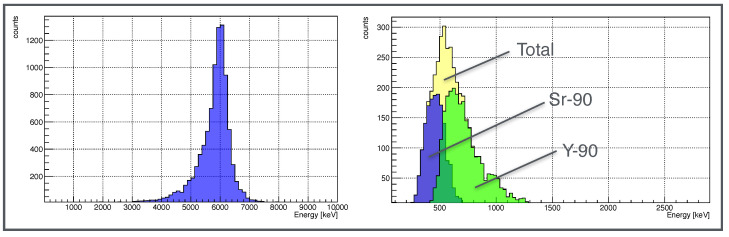
Left canvas: Am-241 α particles energy spectrum. Right canvas: Sr-90 β particles energy spectrum.

The energy spectra obtained for the Am-241 and Sr-90 sources applying the cluster reconstruction algorithm are shown in Figure 6.

The Am-241 α peak shows a low-energy tail due to the energy loss in air and in the detector aluminum backside layer (see Figure 1). The Sr-90 spectrum shape is in good agreement with the one expected for a β emitter. The two subsequent electrons emitted by the source decays, the first in Yttrium (whose endpoint energy $E_{\mathrm{endpoint}}$, defined as the maximum energy transferred to the emitted electron in the β three-body decay, is 546keV) and the second in Zirconium ($E_{\mathrm{endpoint}}=2284\;\mathrm{keV}$, Intensity(%) = 99.98), can be discriminated, as an additional feature, using the number of pixels in the cluster recognized in the 9×9 pixels square area (see Figure 3). In particular, the lower energy tracks (Sr-90) are characterized by a number of pixels less than 9, while the Yttrium electrons tracks are associated with a number of pixels equal to or greater than 9. The Sr-90 energy endpoint shown in the right canvas of Figure 6, is in good agreement with the theoretical value, while the Yttrium endpoint is less than the expected one, because the area of the cluster reconstruction (9×9), optimized for α recognition, cuts the longer β tracks. However, it is possible to scale the algorithm to exploit the potentiality of the technique for β-spectrometry, as described in Section 4 where, by increasing the cluster recognition square area from 9×9 to 13×13 pixels, the Yttrium spectrum is correctly reconstructed.

The β rejection capability of the algorithm was tested using the data acquired with the Sr-90 source: over 12,789 beta events, only 97 were misidentified as α particles, corresponding to a rejection efficiency greater than 99%.

### 3.2. TimePix Radon Exposure: Algorithm Performances

After the algorithm calibration and optimization, the detector response to radon was tested. The TimePix detector was exposed at the INMRI Radon Facility to four different values of radon concentrations (reported in table below), in ‘bare’ configuration.

**Figure 7:**
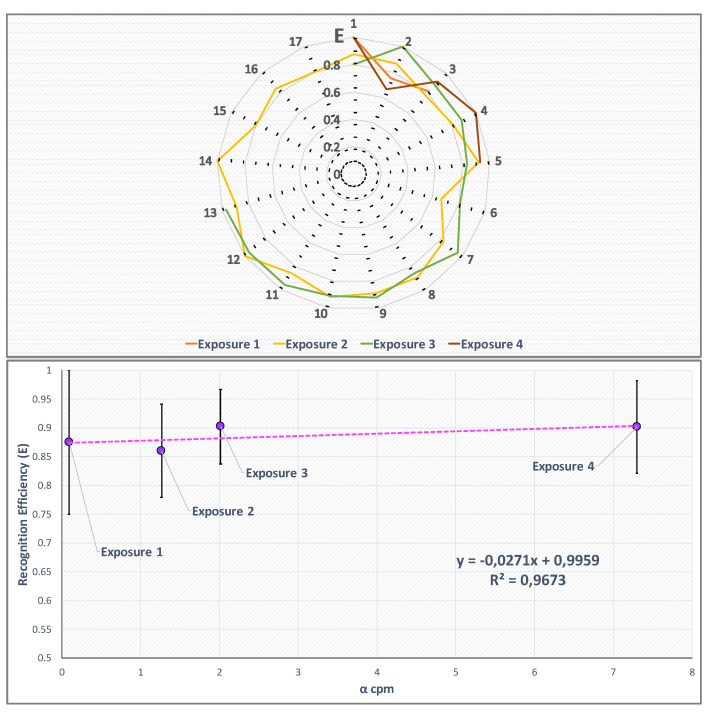
Upper canvas: Recognition efficiency values evaluated according to Equation (4) for the single measurements (*x*-axis shows the number of measurement, *y*-axis the algorithm recognition efficiency E) performed at different radon activity concentration values (different line colors). Lower canvas: mean recognition efficiency value for each exposure versus the mean α counts per minute detected by the TimePix.

The radon activity concentration chosen for the detector characterization, span from very low to quite high values, in order to study the response to typical but also less common concentrations of radon in human habitats. The activity concentration values C(A), reported in Table 1, were measured using the calibrated ALPHAGuard monitor that acquired data simultaneously with the TimePix every 10 min. The TimePix integration time (time to acquire a single frame) was chosen in each exposure to keep the mean pixel hit rate below 1 Hz, so to avoid overpopulated frames. The algorithm performances were evaluated in each exposure by calculating the recognition efficiency while the TimePix response linearity was evaluated in terms of α counts per minute (cpm) in comparison with the activity concentration values measured by the ALPHAGuard detector. The recognition efficiency *E* was evaluated according to the equation:

(4) $$E=\frac{\alpha_{\mathrm{rec}}}{\alpha_{\mathrm{tot}}}$$

where $\alpha_{\mathrm{rec}}$ is the number of α particles recognized by the algorithm in a single frame, and $\alpha_{\mathrm{tot}}$ is the total α clusters number recognised by the frame visual inspection. The result is shown in upper canvas of Figure 7.

The recognition efficiency trend, as function of the α counts per minute (cpm), was studied considering for each radon exposure the *E* mean value (Equation (4)) and the α cpm average value, calculated over the complete exposure time (lower canvas of Figure 7). Uncertainties on the recognition efficiency *E* are evaluated as the standard deviation of the values series (for Exposure 1 and 4 the errors were calculated as the maximum ones). As it is possible to note from the right canvas of Figure 7, the recognition efficiency is almost constant over the considered interval of α cpm.

### 3.3. TimePix Response to Radon

**Figure 8:**
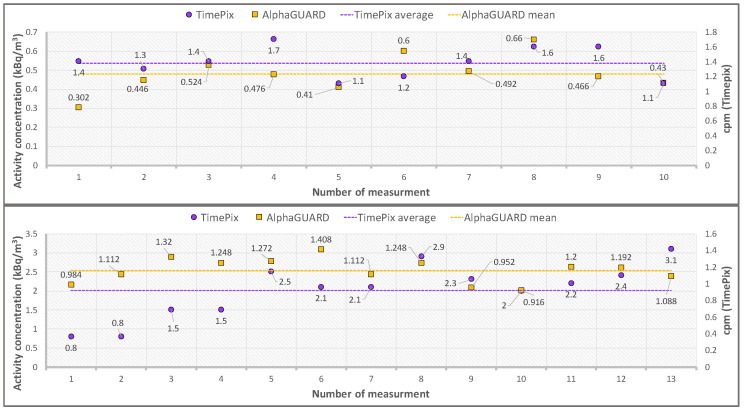
Time series of data acquired by TimePix and the AlphaGUARD are shown for Exposure 2 and 3 (for the run characteristics, see Table 1). Upper canvas—Exposure 2: time series of the radon concentration activity (AlphaGUARD, yellow squares) and the *α* cpm values registered by the TimePix (purple circles). The mean radon activity concentration (dotted yellow line) results equal to 0.48 ± 0.05 kBq/m^3^ and the mean *α* cpm value (purple dotted line) is 1.26 ± 0.21. Lower canvas—Exposure 3: time series of the radon concentration activity (AlphaGUARD, yellow squares) and of the *α* cpm values registered by the TimePix (purple circles). The mean radon activity concentration (dotted yellow line) results equal to 1.16 ± 0.12 kBq/m^3^ and the mean *α* cpm value (purple dotted line) is 2.01 ± 0.48. The statistical errors are evaluated as the standard deviations of the time series.

The TimePix response to radon was evaluated in terms of α cpm detected during the exposures in radon chamber at the ENEA-INMRI radon facility. The cpm (TimePix) and Activity Concentration (AlphaGUARD) time series registered for Exposure 2 and 3 (see Table 1) are shown in Figure 8.

The large volatility, shown by the time series registered by TimePix and AlphaGUARD, is an intrinsic feature of radon measurements performed over small time intervals.

**Figure 9:**
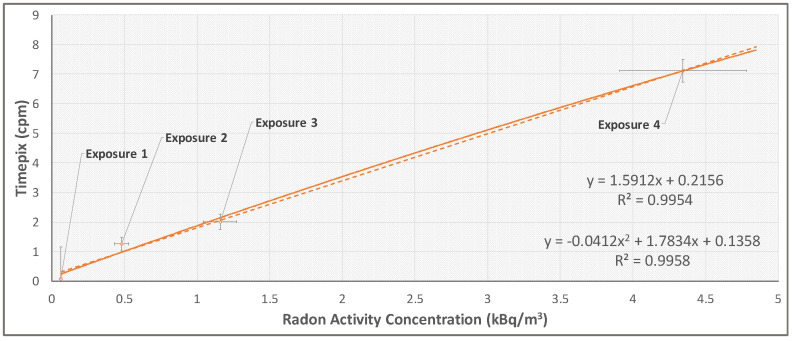
Linearity of the TimePix response. The trendlines equations are shown in figure, where the values of the coefficient R are also reported.

To overcome this problem and study the TimePix response linearity at different radon concentration values, the α cpm and radon activity concentration values averaged over the total time exposure were considered. The result is shown in Figure 9.

The TimePix response to different radon concentrations activity in ‘bare’ configuration shows good linearity, with a linear fit correlation coefficient higher than 0.99. The square correction was also evaluated by fitting the data using a square function of the type $y=ax^{2}+bx+c$. The best fit value of the square term coefficient *a* is consistent with zero value within the associated error.

### 3.4. Estimation of the Virtual Enclosure Volume

The Virtual Enclosure volume (VEV, shown in the right canvas of Figure 4), was estimated for the TimePix detector in the ‘bare’ configuration using the Am-241 source. The dimension of the VEV, depending on the maximum distance that an α particle can travel without losing all its energy in medium ionization, varies as a function of the particle energy and also of the air temperature and pressure.

**Figure 10:**
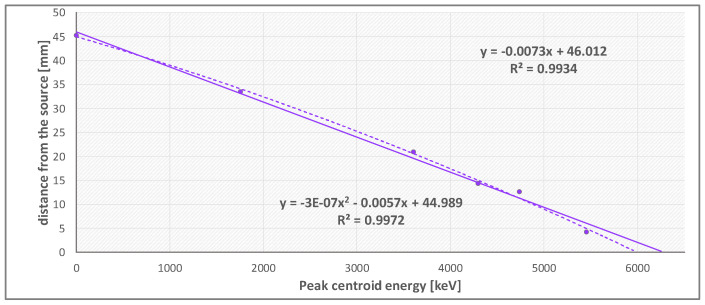
Measurements of the Am-241 α peak centroid as a function of the distance between the source and the detector and the linear and a square fit used to interpolate the distance value.

The α particles of the Am-241 source, whose energy (${\bar{E}}_{\alpha }≃5.5$ MeV) can be considered representative for Rn-222 ($E_{\alpha }=5.49$ MeV) Po-218 ($E_{\alpha }=6.00$ MeV) and Po-210 ($E_{\alpha }=5.30$ MeV) radionuclides at given conditions of temperature ($28{\;}^{\circ }C$) and pressure (1015mBar). Six measurements of the *α* peak centroid (deposited energy) were performed by placing the Am-241 source at a variable distance above the detector, acquiring 200 frames with an integration time of 3 s for each distance. Since the radon around the detector in the ‘bare’ measurement configuration can be ideally represented as an infinite number of planar sources stacked on top of each other above the sensor, the presented measurement method is a discrete approximation of the ideal situation. The result is shown in Figure 10.

The data were fitted with a linear and a square function, providing a correlation coefficient higher than 0.99 for both. The final result, in terms of the maximum distance traveled by *α* particle which define the VEV dimensions, is 46 ± 2 mm, calculated as the mean value between the linear and the square models results. This approach was needed because the planar source dimensions (⌀ = 50 mm) are not negligible with respect to the detector dimensions at small distances, so the point-like source approximation is not directly applicable. The linear and the square functions correspond to the minimum and maximum values used to interpolate the mean value. The related uncertainty value does not include the systematic contribution, which will be evaluated in the next dedicated measurement campaign (see Section 4).

## 4. Discussion and Conclusions

The results presented in this work show the feasibility of exploiting a pixelated silicon detector to measure the radon concentration through its *α* emitting progeny in ‘bare’ configuration, thanks to the application of the novel algorithm specifically developed. A TimePix detector was used to test the algorithm performances in terms of *β* rejection capability (number of *β* misidentified as *α*) and of recognition efficiency (number of *α* recognized over the total number of *α* particles identified by visual inspection). The *α*/*β* discrimination, based on the pixel cluster geometrical features and the energy dispersion around the seed, allows to have a linear detector response over the considered interval of radon activity concentration.

The possibility to efficiently identify the *α* particles without resorting to spectroscopic methods opens to several possibilities for radon continuous monitoring with silicon pixelated detectors. The simple measurement configuration and the small dimensions of the detector permit to ‘locally’ measure in real-time mode the radon concentration through its *α* progeny, allowing to map the indoor radon and eventually implementing adequate countermeasures. Such capability could be possibly integrated into a home or office automation system, where a single sensor can be used to monitor a typical room up to 30 m^2^ with the possibility to double the number of sensors for a larger room or when radon concentration values are particularly high. At the same time the detector, thanks to its handy and small dimensions, can be used in ‘bare’ configuration for inspecting in daily surveys also areas generally hard to reach with other detectors, currently available on the market, which have larger dimensions.

In principle, a similar approach could be implemented on commercial pixelated silicon detectors like CMOS and CCD devices. These sensors, commonly used in cameras with good performance and available on the market at low cost, have a pixelated matrix structure that can be potentially used for this kind of measurements. In particular, if a Single-Board Computer (SBC) today available on the market is used to drive commercial cameras containing such sensors, it will be possible to reduce costs for a single radon sensor below hundred euros. However, in this case, further studies are still needed to investigate how to overcome some of the hardware issues, in particular how the ambient light can affect a measurement performed with a camera sensor.

**Figure 11:**
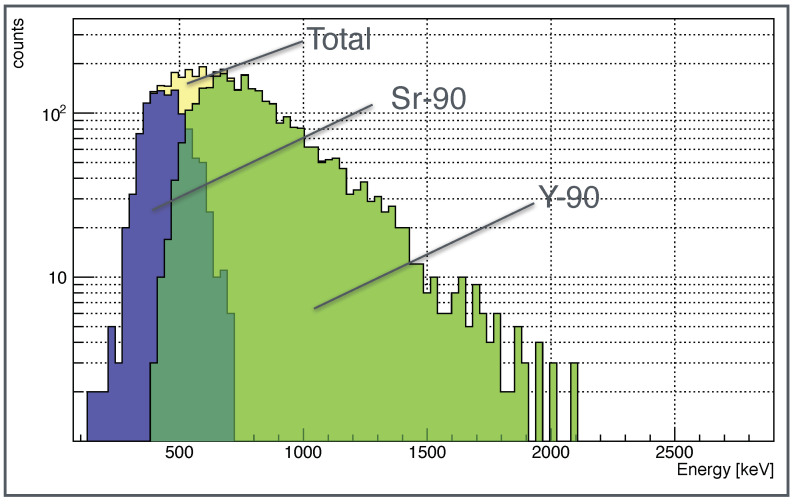
Sr-90 source spectrum resulted by the algorithm optimization to measure the Y-90 electrons. The clusters reconstruction area is increased from 9 × 9 to 13 × 13 pixels.

A further feature of the algorithm is its scalability, which permits to optimize it as a function of the radionuclide to be measured, allowing the detector to be used also as *α* and *β* spectrometer. Once *α* or *β* tracks have been identified it is possible to study their energy spectrum. The results obtained for Sr-90 source, with the algorithm optimized to measure electrons from Yttrium are shown in Figure 11.

In this case, the area used to reconstruct the cluster is incremented from 9 × 9 to 13 × 13 pixels. As it is possible to note, the Yttrium histogram shape shows a good agreement with the source *β* endpoint energy value. This result opens the possibility to use this kind of detector to monitor other radionuclides in air in *Continuous Air Monitoring* (CAM) configuration. In this application, the device can be coupled to a collection filter where the air is continuously flushed. This kind of detection system is of fundamental importance in installations where there is the risk of air contamination, like nuclear reactors or radiopharmaceutical facilities. In this case, the algorithm should be optimized according to the radionuclide to measure.

Future developments of the presented work will involve TimePix detector as well as commercial silicon pixelated sensors read by SBCs. Concerning the TimePix performance for radon measurements, future developments will concern detailed simulations of the detector to evaluate the total efficiency of the system and, in particular, to estimate the Minimum Detectable Concentration (MDC) value, since this quantity provides fundamental information about the decision threshold for radiological measurements. Moreover a cross-calibration procedure will be performed to evaluate the TimePix response in terms of radon activity concentration at the ENEA Metrological Institute (INMRI) radon facility, using a large set of radon concentration values and also different values of pressure, temperature, and relative humidity. These studies, which will allow evaluating also the algorithm efficiency as a function of the pixel hit rate, will complete characterization of TimePix to provide reliable results for radon measurement that can be used for risk evaluation in radiological applications.

Concerning a possible roll-out of the presented solution, which implies implementing commercial pixelated silicon sensors and their integrated electronics read by SBCs, several issues need to be investigated on both software and hardware sides. Among the issues that will be addressed in further studies there are: data conversion, since commercial hardware generally produce data in image format that should be converted in a usable format for the algorithm; detector radiation response and the contributions of the external variables (in particular the ambient light); characterization of the system for radon exposure, including also the usable range in terms of temperature, pressure and relative humidity. Only after these key points, and eventually other ones that could come out, will be addressed, the method roll-out can be properly considered and effectively planned on a scale which can positively impact public health.

## Figures and Tables

**Table 1:** Exposure run parameters used for the TimePix characterisation at INMRI radon facility. The average radon activity concentration C(A) values in Bq/m^3^ were measured using the calibrated ALPHAGuard monitor. The TimePix parameters set for the different exposures are reported below the radon activity concentration values.

	Exposure 1	Exposure 2	Exposure 3	Exposure 4
C(A) [Bq/m^3^]	64 ± 6	481 ± 50	1158 ± 120	4345 ± 430
Total exposure time [min]	120	170	120	10
Integration time [min]	60	10	10	2
Number of frames acquired	2	17	12	5
mean pixel hit rate [Hz]	0.18 ± 0.01	0.68 ± 0.03	0.74 ± 0.04	0.21 ± 0.04

## Data Availability

Not applicable.

## Abbreviations

WHO	World Health Organization
ISS	International Space Station
EFT-1	Orion Exploration Flight Test 1
ASIC	Application Specific Integrated Circuit
RDP	Radon Decay Product
CPM	Counts Per Minute
VEV	Virtual Enclosure Volume
CMOS	Complementary Metal-Oxide-Semiconductor
MDC	Minimum Detectable Concentration
CCD	Charge Coupled Device
SBC	Single-Board Computer
